# *Leptospira* Lipid A Is a Potent Adjuvant That Induces Sterilizing Immunity against Leptospirosis

**DOI:** 10.3390/vaccines11121824

**Published:** 2023-12-06

**Authors:** Vivek P. Varma, Mohammad Kadivella, Sridhar Kavela, Syed M. Faisal

**Affiliations:** 1 Laboratory of Vaccine Immunology, National Institute of Animal Biotechnology, Hyderabad 500032, India; vivek@niab.org.in (V.P.V.); mohammadk@niab.org.in (M.K.); sridharkavela@chaitanya.edu.in (S.K.); 2 Graduate Studies, Manipal Academy of Higher Education, Manipal 576104, India; 3 Regional Centre for Biotechnology, Faridabad 121001, India

**Keywords:** LigA, lipid A, adjuvant, vaccine, MPLA, alum, leptospirosis

## Abstract

Leptospirosis is a globally significant zoonotic disease. The current inactivated vaccine offers protection against specific serovars but does not provide complete immunity. Various surface antigens, such as *Leptospira* immunoglobulin-like proteins (LigA and LigB), have been identified as potential subunit vaccine candidates. However, these antigens require potent adjuvants for effectiveness. Bacterial lipopolysaccharides (LPSs), including lipid A, are a well-known immunostimulant, and clinical adjuvants often contain monophosphoryl lipid A (MPLA). Being less endotoxic, we investigated the adjuvant properties of lipid A isolated from *L. interrogans* serovar Pomona (PLA) in activating innate immunity and enhancing antigen-specific adaptive immune responses. PLA activated macrophages to a similar degree as MPLA, albeit at a higher dose, suggesting that it is less potent in stimulation than MPLA. Mice immunized with a variable portion of LigA (LAV) combined with alum and PLA (LAV-alum-PLA) exhibited significantly higher levels of LAV-specific humoral and cellular immune responses compared to alum alone but similar to those induced by alum-MPLA. The adjuvant activity of PLA resembles that of MPLA and is primarily achieved through the increased recruitment, activation, and uptake of antigens by innate immune cells. Furthermore, like MPLA, PLA formulation establishes a long-lasting memory response. Notably, PLA demonstrated superior potency than MPLA formulation and provided sterilizing immunity against the leptospirosis in a hamster model. Overall, our study sheds light on the adjuvant properties of *Leptospira* lipid A and offers promising avenues for developing LPS-based vaccines against this devastating zoonotic disease.

## 1. Introduction

Leptospirosis, a zoonotic disease, remains a significant global public health concern, becoming even more of a threat due to the impacts of climate change and global warming, which have escalated its prevalence. Annually, there are approximately one million reported cases of human leptospirosis, resulting in an estimated 60,000 fatalities [1]. Despite the availability of a broad spectrum of antibiotics, their effectiveness diminishes when the bacteria infiltrate vital organs and cause substantial damage, often due to delayed diagnosis. Vaccination is a cost-effective and secure preventive measure to combat this disease. However, the current killed vaccine only provides short-term immunity specific to certain serovars and fails to prevent bacterial shedding through urine. Efforts to develop live attenuated vaccines capable of inducing long-term protection have resulted in the creation and testing of various virulence-attenuated mutants [2,3]. Immunization with a mutant of the flagellar protein (FcpA) has demonstrated cross-protection against different pathogenic *Leptospira* species, and immunization with the LPS mutant strain M1352 led to an 80% reduction in renal colonization upon exposure to the same serovar [2]. These attenuated vaccines despite inducing robust cross-protection, have fallen short of providing sterilizing immunity, and their capacity to generate a long-lasting protective response has not been tested. Therefore, it is imperative to develop potent vaccines capable of eliciting sterilizing immunity against multiple serovars.

Subunit vaccines represent a cutting-edge generation of candidates prioritizing safety by utilizing components from the outer membranes of microbes, such as lipopolysaccharides, surface proteins, glycoproteins, and toxoids [4,5]. Various outer membrane and surface proteins from *Leptospira* have been explored as potential subunit vaccine candidates and have demonstrated varying degrees of protection in animal models [6]. Among these proteins, *Leptospira* immunoglobulin-like protein A (LigA), specifically its C-terminal or variable region (LAV), has emerged as an exceptionally promising candidate, supported by the findings of several researchers [7,8]. Adjuvants are crucial in enhancing vaccine efficacy by boosting the immune response without directly contributing to antigen-specific protection [9]. Several surface proteins from *Leptospira* have been tested in combination with potent adjuvants like Freund’s adjuvant, liposomes, PLGA-microparticles, xanthan gums, as well as emulsions like AddaVax and Emulsigen-D. These combinations have provided varying degrees of protection, ranging from 50% to 70%. However, none of the formulations have provided sterilizing immunity [7,10,11,12,13]. It is important to note that, except alum, all these adjuvants are in the preclinical stage. Fortunately, a new generation of clinical adjuvants, including Montanide, MF59 and Adjuvant Systems (AS03, AS04), have been developed, showing strong potential in enhancing antigen-specific immune responses and protective efficacy [14]. For instance, AS04 combines monophosphoryl lipid A (MPLA), a TLR4 agonist, with an aluminum salt, and has been tested in vaccines against HPV and HBV [15]. Our recent study has demonstrated that LAV formulated with AS04 leads to an enhanced immune response and improved protection in a hamster model of leptospirosis [16]. These findings underscore the potential of AS04 or similar adjuvant formulations in developing a potent vaccine against leptospirosis.

Lipopolysaccharide (LPS) is a major antigen found in Gram-negative bacteria [17]. It is a stimulant of TLR4, promoting the activation and maturation of antigen-presenting cells (APCs). However, LPS’s toxicity has hindered its use as an adjuvant in human vaccines [18]. The removal of phosphate from lipid A, derived from the LPS of *Salmonella minnesota*, has mitigated its toxicity, creating a less toxic derivative known as monophosphoryl lipid A (MPLA) [19]. While both LPS and MPLA are recognized by TLR4, MPLA triggers a less inflammatory pathway involving a TRIF adaptor [20]. When MPLA is formulated with alum (AS04), it can enhance the antigen-specific immune response and modulate it toward a mixed TH1/TH2 or a polarized Th1 cell response. In contrast, alum primarily elicits a skewed Th2 response [21,22]. Most of AS04′s adjuvant activity is attributed to the immunostimulatory properties of MPLA, although alum aids in prolonging this stimulation [22]. *Leptospira* LPS, a significant antigen, induces protective immunity in animal models [3]. It is recognized as being naturally less toxic and atypical, signaling through TLR4 and TLR2 [23]. Considering the crucial roles of TLR2 and TLR4 in protecting against leptospirosis and recognizing the potent adjuvant effects of MPLA, it was of interest to assess the adjuvant potential of the naturally less toxic *Leptospira* LPS, specifically lipid A, against leptospirosis [16,24].

In our current investigation, we isolated lipid A from *Leptospira interrogans* serovar Pomona (PLA) and conducted experiments to evaluate its immunostimulatory effects on mouse macrophages. Subsequently, we examined the adjuvant properties of PLA in mice, initially focusing on its ability to enhance the immune response to a model antigen, Ovalbumin (OVA), and then extending our assessment to a *Leptospira* surface antigen (LAV). We compared the immune responses elicited by these antigens when formulated with either alum alone or in combination with MPLA or PLA. To better understand how PLA functions as an adjuvant, we investigated its capacity to induce a localized inflammatory response and establish lasting immune memory. Finally, we evaluated the immune response and protective effectiveness of the PLA-based formulation in a hamster model of leptospirosis.

## 2. Material and Methods

### 2.1. Study Design

In this study, our primary objective was to investigate *Leptospira* lipid A’s immunostimulatory and adjuvant properties, explicitly focusing on lipid A derived from the pathogenic *Leptospira interrogans* serovar Pomona (referred to as PLA). We compared PLA activity alongside MPLA, a well-established stimulatory agent and clinical adjuvant. To assess PLA’s stimulatory potential, we utilized mouse macrophages to measure their capacity to induce pro-inflammatory cytokines and maturation markers. Furthermore, we examined PLA’s capability to enhance antigen-specific humoral and cellular immune responses. We initially combined PLA with a model antigen, Ovalbumin (OVA), and a 2% Alhydrogel adjuvant. This allowed us to determine the optimal dosage of PLA. Subsequently, we incorporated a variable proportion of *Leptospira* immunoglobulin-like protein A (LAV) into the formulation. The immune response against various formulations was analyzed in mice following established methodologies.

To gain insights into the mechanism of action and identify critical innate immune cell mediators, we administered mice with PLA- or MPLA-based formulations containing a fluorescent antigen (LAV). Using advanced techniques such as flow cytometry and RT-PCR, we evaluated immune cells’ recruitment, antigen uptake, and their activation status in draining lymph nodes. Our research also assessed the long-term memory response induced by PLA-based formulations. We monitored LAV-specific antibody levels for up to 24 weeks and subsequently analyzed B and T cell memory responses following in vitro stimulation with LAV as a recall antigen.

Finally, we critically evaluated the efficacy of the PLA-based vaccine formulation against a challenge with virulent *Leptospira* in a hamster model. This evaluation involved assessing the survival rate of the hamsters, quantifying the bacterial load in their organs, and examining any lesions in infected organs through a histopathological examination.

### 2.2. Chemicals and Reagents

Unless specifically mentioned otherwise, most of the chemical and cell culture reagents used in this study were sourced from Sigma-Aldrich. St. Louis, MO, USA. We acquired ELISA kits from R&D Biosystems, while BD Biosciences supplied the antibodies for flow cytometry. For the MPLA and 2% Alhydrogel^®^ (alum), we procured them from InvivoGen, San Diego, CA, USA.

### 2.3. Animals and Housing Conditions

The animal experiments were conducted in accordance with the guidelines set forth by the Committee for the Purpose of Control and Supervision of Experiments on Animals (CPCSEA), Government of India. They were approved by the Institutional Animal Ethics Committee (IAEC) under the reference numbers IAEC/2019/NIAB/18/SF and IAEC/2020/NIAB/11/SF. Female C57BL/6J mice, aged 5 to 6 weeks, were initially procured from the Jackson Laboratory in the USA. These mice were subsequently bred and housed in the Animal Resource and Experimental Facility at the National Institute of Animal Biotechnology (NIAB). Male golden Syrian hamsters were sourced from Charles River and were bred and maintained at Jeeva Life Sciences Pvt. Ltd. in Hyderabad, where the experiments were conducted.

All animals were kept in a controlled environment with a 12 h light and 12 h dark diurnal lighting cycle, maintained at a temperature of 23–24 °C and a humidity level of 45%. Standard pathogen-free conditions were ensured, and the animals had unrestricted access to food and water throughout the study. To monitor their well-being, the mice and hamsters were visually checked twice daily for vital signs and any signs of illness. After the study, the animals were humanely euthanized using 5% isoflurane following the CPCSEA’s Euthanasia Guidelines. Confirmation of death was verified through cervical dislocation, performed two minutes after breathing cessation.

### 2.4. Antigen Preparation

The OVA was reconstituted in endotoxin-free water (Millipore Sigma, Burlington, MA, USA) at a 1 mg/mL concentration and then stored at −20 °C. The LAV was purified using a previously established method [16]. Briefly, *E. coli BL21 (DE3)* cells carrying the expression plasmid pET28a-LAV were cultured in LB broth supplemented with kanamycin (40 μg/mL). Protein expression was induced by adding 1 mM of isopropyl β-D-1-thiogalactoside (IPTG). After induction, the cells were harvested through centrifugation at 8000 rpm for 5 min. Subsequently, cell lysis was achieved using a lysis solution (composed of 150 mM of NaCl and 100 mM of Tris-HCl at pH 8.0), followed by a sonication step. Debris was removed via centrifugation, and the resulting supernatant was subjected to affinity chromatography using a Ni-NTA bead column from GE Healthcare. The eluted protein was dialyzed against 1 × PBS and passed through Detoxi-Gel^TM^ from Pierce (Waltham, MA, USA) to eliminate *E. coli* LPS contamination. The protein’s endotoxin level was assessed using the Limulus amoebocyte lysate (LAL) assay kit (Thermo Fischer, Waltham, MA, USA) and found to be below 0.011 EU/mL. The protein purity and size were evaluated through SDS-PAGE, and its concentration was determined using the Bradford reagent from Sigma-Aldrich.

### 2.5. Leptospira Culture

The pathogenic variant of *L. interrogans* serovar Pomona was grown in a liquid Ellinghausen–McCullough–Johnson–Harris (EMJH) medium at a temperature of 28 °C. The strain underwent a series of passages in the golden Syrian hamsters to enhance its virulence, after which it was isolated from the infected kidneys. The virulent Leptospires were subsequently kept in a semi-solid culture.

### 2.6. Lipid A Isolation and TLC

Lipid A was extracted from *Leptospira* using a previously published method with slight modifications [25]. In brief, logarithmic growth phase cultures of *Leptospira interrogans* serovar Pomona were collected, washed twice with DPBS, and then resuspended in a Bligh–Dyer mixture consisting of chloroform, methanol, and water at a ratio of 1:2:0.8 (*v*/*v*). The mixture was agitated for 20 min at room temperature. Following this, the cellular fragments were separated through centrifugation at 2000 rpm for 20 min, and the lipid A component was isolated from the lipopolysaccharide (LPS) by subjecting it to an hour of boiling in a hydrolysis buffer containing 50 mM of sodium acetate and 1% sodium dodecyl sulfate, adjusted to a pH of 4.5. After cooling to room temperature, the samples underwent a second extraction step using a two-phase Bligh–Dyer mixture composed of chloroform, methanol, and water at a ratio of 2:2:1.8 (*v*/*v*/*v*). The samples were centrifuged at 2000 rpm for 20 min to separate the phases, and the lower phase containing lipid A was transferred and subsequently dried using a rotary evaporator. The quality of the extracted lipid A was evaluated using silica thin-layer chromatography (TLC) as per the previously described method [25]. Briefly, 10 µL of lipid A dissolved in a mixture of chloroform and methanol (4:1, *v*/*v*) was spotted onto a silica plate using a microcapillary glass pipette and allowed to air-dry for 15 min. The plate was then placed in a tank pre-equilibrated with the mobile phase (chloroform/pyridine/88% formic acid/water; 50:50:16:5 *v*/*v*). Once the solvent front reached the top of the plate, it was removed, air-dried, and immersed in a 10% sulfuric acid–ethanol solution. Subsequently, the plate was air-dried in a fume hood and visualized by heating it on a 250 °C ceramic hot plate until the lipid A became charred and visible.

### 2.7. Cell Stimulation Assay

The isolated lipid A was carefully weighed and dissolved in endotoxin-free water (MilliporeSigma, Burlington, MA, USA) to achieve a 1 mg/mL concentration. This dissolution process was carried out by placing it in a water bath sonicator (Cole-Parmer, Vernon Hills, IL, USA) for 10 min at a temperature of 37 °C. RAW264.7 cells (obtained from ATCC, Manassas, VA, USA) were seeded into 24-well plates and then stimulated with various concentrations of PLA (0.1/0.5/1/2/5/10/20/50 µg/mL) or MPLA (0.5/1/2 µg/mL) for 24 h at 37 °C, 5% CO_2_. Following this stimulation, the cytokine IL-6 was measured in the culture supernatant using a Sandwich ELISA kit (R&D Systems, Minneapolis, MN, USA) following the manufacturer’s instructions.

### 2.8. Vaccine Formulation

The test and control formulations were prepared on the dosing day using the following method: The optimal dosage (20 or 40 µg/animal) for inducing in vivo adjuvant activity of PLA was determined with Ovalbumin. Various vaccine formulations were created by combining 100 µg of alum with MPLA (5 µg) or PLA (20 or 40 µg), along with an equal volume of DPBS containing the OVA or LAV antigen (10 µg for the first immunization, and 5 µg for the booster) in a 1:1 ratio. In contrast, formulations for hamster immunization were prepared with LAV (50 µg for the first immunization and 25 µg for the booster) in alum, along with MPLA (20 µg) or PLA (80 µg). To prepare Heat Killed Leptospira (HKL), the log-phase culture of Pomona was counted using the Petroff-Hausser Counter. The cells were washed twice with DPBS, heat-inactivated at 56 °C for 30 min, and then suspended in DPBS at a concentration of 2 × 10^9^ cells/mL, mixed with 100 µg of Alhydrogel in a 1:1 volume ratio.

### 2.9. Animal Immunizations and Sample Collection

Six male C57BL/6 mice per group were subcutaneously immunized with various vaccine formulations, with a total volume of 100 μL per animal. These mice received a booster dose as described above. Blood samples were taken from the mice before immunization (pre-bleed), one week after the booster, and on the 28th day when the animals were euthanized. Spleens and lymph nodes were collected for further analysis. To assess the long-term immune response and the development of immunological memory, the mice received additional blood draws every other week for up to 24 weeks (approximately 6 months) after the booster. Some mice were euthanized with or without an additional booster, and blood, spleens, and lymph nodes were collected for memory response analysis. Tissues from the injection site were collected 4 and 24 h after immunization and preserved in an RNAprotect solution (Qiagen, Hilden, Germany).

Male golden Syrian hamsters, aged 4 to 5 weeks, were grouped and subcutaneously immunized with different formulations: PBS, heat-killed/inactivated bacterin (HKL; 10^9^), LAV-alum LAV-alum-MPLA, or LAV-alum-PLA. The total administered volume was 200 μL, followed by a booster dose on the 21st day with 25 µg of the antigen. Before immunization, the hamsters were anesthetized using an intraperitoneal injection of ketamine (10 mg/mL) and xylazine (1 mg/mL) at 100 µL per 130 g of body weight. Blood samples were collected on days 0, 21, and 35, and the hamsters were euthanized on day 35. Spleens were collected to assess the immune response specific to the LAV antigen.

### 2.10. ELISA for Serum Antibody Levels

We determined the antibody levels in serum collected from the mice and hamsters using an ELISA assay, following a well-established standard protocol as previously described [26]. In brief, 96-well microtiter plates from Nunc, Denmark, were used for the assay. These plates were coated with antigens, specifically OVA or LAV (at a concentration of 100 ng/well for mice and 200 ng/well for hamster experiments) or PLA (500 ng/well) in a 0.1 M bicarbonate buffer. The plates were then incubated overnight at 4 °C. The following day, we washed the plates three times using 1 × PBS with a 0.05% Tween 20 (PBST) solution and subsequently blocked them with 1% BSA for 1 h at room temperature. After another round of washing, we added 100 µL/well of diluted serum (ranging from 1:100 to 1:100,000 in PBST) and allowed them to incubate for 2 h at 37 °C in a humid chamber. Following this incubation, the plates were rewashed, and 100 µL/well of HRP-conjugated secondary antibodies were added (diluted at 1:6000). We used goat anti-mouse secondary antibodies specific to IgG, IgG1, IgG2c, or IgA for mice. For the hamsters, we used mouse anti-hamster antibodies against total IgG, IgG1, or IgG2/3. These were incubated for 1 h at room temperature. After another round of washing (five times), we added 100 µL/well of the TMB substrate and left it at room temperature, shielded from direct light, for 20 min. The enzymatic reaction was halted by adding 50 µL of 2N H2SO4, and we measured the optical density at 450 and 540 nm using an ELISA reader from Perkin Elmer.

### 2.11. Cell Proliferation and Cytokine Estimation

Lymphocyte proliferation was assessed using splenocytes from different groups of immunized mice and hamsters. To determine proliferation, the cells were stimulated with recall antigens (OVA or LAV) and then counted after 48–72 h, as previously described [16]. In brief, splenocytes (1 × 10^5^ cells/well) were plated in a 24-well plate and exposed to varying concentrations (1, 2.5, and 5 µg/mL) of OVA or LAV for 48 to 72 h. Cell counting was performed using the trypan blue size exclusion method. The levels of IL-4 and IFN-γ released into the culture supernatant after 48 h were quantified using sandwich ELISA kits (R&D Systems), following the manufacturer’s instructions. Lymphocytes from various groups of hamsters were stimulated with varying concentrations (1, 2, and 10 µg/mL) of LAV for 48 to 72 h. Subsequently, the cells were collected, RNA was isolated, and the expression of IL-4 and IFN-γ was analyzed through RT-PCR.

### 2.12. Flow Cytometry

We initiated the experiment by seeding RAW264.7 cells in 12-well plates at a density of 0.1 × 10^6^ cells per well to investigate the impact of PLA treatment on macrophage activation. These cells were exposed to either MPLA (1 µg/mL) or lipid A (2 or 5 µg/mL) for 24 h. After treatment, the cells were washed twice with prechilled PBS followed by blocking with rat anti-mouse CD16/CD32 (FC antibody; 553141, BD Biosciences, Franklin Lakes, NJ, USA) in a solution of PBS containing 0.5% (*w*/*v*) BSA and 2% (*v*/*v*) FBS for 30 min. Following this, the cells underwent three additional washes and were subsequently incubated on ice with the following antibodies obtained from BD Biosciences: PerCp Cy5.5 rat anti-mouse I-A/I-E (MHC-II; 562363); APC hamster anti-mouse CD80, 560016; PE rat anti-mouse CD86, 560601, and BV421 rat anti-mouse CD40, 562846. Unbound antibodies were removed through three washes, and the cells were fixed using 1% paraformaldehyde.

To investigate the adjuvant mediated recruitment, antigen uptake, and activation status of APCs, we utilized LAV labeled with Alexa Fluor™ 488 (Invitrogen-A10235, Waltham, MA, USA) formulated with various adjuvants. These formulations were injected, and draining lymph nodes (DLNs) were isolated at 4 and 24 h post-injection. The DLN cells were washed and subjected to Fc receptor blocking at 4 °C for 30 min. Subsequently, these cells were stained with BV-421 hamster anti-mouse CD11c (562782) and PerCp Cy5.5 rat anti-mouse I-A/I-E (MHC-II; 562363) for dendritic cells; BV510 rat anti-mouse CD11b (562950) and PE-CF 594 Rat anti-Mouse F4/80 (565613) for monocytes/macrophages; BV510 rat anti-mouse CD11b (562950) and BV605 rat anti-mouse Ly6C (563011) for monocytes; BV510 rat anti-mouse CD11b (562950) and PE-Cy7 rat anti-mouse Ly6G (560601) for neutrophils; and APC hamster anti-mouse CD80 (560016), PE rat anti-mouse CD86 (560601), and PerCp Cy5.5 rat anti-mouse I-A/I-E (MHC-II; 562363) to identify specific cell types and assess their activation status.

In a separate series of experiments to determine the T cell population, splenocytes from different groups obtained on the 28th day or at 24th and 25th week post-immunization were stained with antibodies from BD Biosciences, including rat anti-mouse FITC-CD3 (555274), BV421-CD4 (562891), PE-CD8a (567630), APC-Cy 7-CD44 (560568) and BV650-CD62L (564108). Similarly, splenocytes obtained from various groups of hamsters (on the 35th day) were stained with eBiosciences APC rat anti-mouse CD4 (17-0041-82)/PE mouse anti-rat CD8b (12-0080-82). Following staining, the cells were washed and fixed with 1% paraformaldehyde. Data acquisition (100,000 events/sample) was performed using the BD LSRFortessa™ Cell Analyzer, and subsequent analysis was carried out using the FlowJo software (BD Biosciences, Franklin Lakes, NJ, USA).

### 2.13. Generation of Bone Marrow-Derived DCs

The dendritic cells derived from bone marrow were prepared using a previously established protocol [16]. In brief, bone marrow cells were collected from mice and cultured in 100 mm dishes at a density of 1 × 10^7^ cells per dish. These cells were cultured in a complete DMEM medium supplemented with GM-CSF (20 ng/mL; Preprotech, Thermo Fischer Scientific, Cranbury, NJ, USA) and IL-4 (5 ng/mL; Preprotech). To replenish the culture, half of the medium in each dish was replaced with fresh, complete DMEM media containing GM-CSF (40 ng/mL) and IL-4 (10 ng/mL) on the second and seventh days. On the ninth day, non-adherent cells were removed, while the adherent cells were gently scraped off. The cell count and viability were assessed, and these cells were subsequently utilized for specific assays.

### 2.14. CTL Assay

Antigen-specific cytotoxic T cells (CTLs) generated on the 28th day post-immunization were assessed through an LDH-based cytotoxicity assay, as previously detailed [16]. To outline the procedure briefly, we prepared a suspension of 3 × 10^7^ splenocytes in 10 mL of complete RPMI medium supplemented with 0.2 ng/mL of IL-2, which was then cultured in 25 cm^2^ tissue culture flasks. After 5 days of upright maintenance, the non-adherent cells were collected and utilized as effector (E) cells.

In parallel, dendritic cells (DCs; 5 × 10^5^) were seeded into 12-well plates and exposed to 2 µg/mL of OVA, LAV, or BSA (Bovine Serum Albumin) for 16 h at 37 °C with 5% CO_2_. These treated DCs were employed as target cells (T) and were further treated with mitomycin C (50 μg/mL) for 45 min. The E-cells were counted and then incubated with the target cells (T), creating different E/T ratios (10:1, 25:1, and 50:1), followed by an additional 5 h incubation at 37 °C with 5% CO_2_. In this setup, DCs pulsed with OVA or LAV acted as specific target cells, while DCs pulsed with BSA represented non-specific target cells. The cytotoxicity evaluation was conducted using the LDH assay kit (Cytotox 96, Promega), focusing on assessing the specific lysis of target cells by CTLs, as previously outlined [27]. The maximum release was achieved by lysing DCs with Triton X-100 at a final concentration of 1% (vol/vol), while the LDH released by untreated cells was considered the spontaneous release. Cytotoxicity was calculated using the following formula:

$$\%\;specific\;lysis=\frac{\left(experimental\;release-spontaneous\;release\right)}{\left(maximum\;release-spontaneous\;release\right)}$$

### 2.15. Lymph Node Sectioning and Immunofluorescent Staining

The inguinal draining lymph nodes from different groups were harvested on the 28th day and processed following previously established protocols. In brief, the lymph nodes were initially fixed in a 4% paraformaldehyde (PFA) solution for one hour, then gradually dehydrated using sucrose solutions of 10%, 20%, and 30% at 4 °C. Afterward, the lymph nodes were cryopreserved in liquid nitrogen within cryomolds and stored at −80 °C until further processing.

A Leica Cryostat (Leica Geosystems, Aarau, Switzerland) was employed to create 12 μm thick slices for sectioning. These sections were rehydrated in phosphate-buffered saline (PBS; pH 7.4) for three hours and then blocked using a solution of 5% BSA and 0.05% Tween 20 in PBS for one hour. Immunostaining was performed using the following antibodies: Rat anti-mouse FITC-conjugated CD3 (555274, BD Biosciences, 1:300), rat anti-mouse Per-CP-conjugated CD45R (B220; 130-102-815, Miltenyi Biotec, San Jose, CA, USA, 1:100), rat anti-mouse PE-conjugated GC B-Cell marker (GL-7; 561530, BD Biosciences, 1:200), and rabbit anti-mouse Follicular Helper T cell marker CXCR5 (ab254415, Abcam, 1:500). This incubation was carried out overnight in a moist chamber at 4 °C. Following immunostaining, the sections were thoroughly washed five times with PBS and then incubated with the secondary antibody, goat anti-rabbit Alexa Fluor^®^ 405-conjugated IgG H&L (ab175652, Abcam, 1:300), for two hours at room temperature. The antibody-stained sectioned slides were mounted using the VECTASHIELD^®^ Antifade mounting medium from Vector Laboratories. Imaging was performed using a Carl Zeiss Axio Scope VII fluorescent microscope with a 20× Plan Apochromat 0.45 NA objective and an EXFO X-Cite metal halide light source. The images were captured in tile-scanning mode using a Hamamatsu ORCA-ER CCD camera, and subsequently, the Zen Blue software (Version 3.7, Carl Zeiss, Jena, Germany) was used for image processing and stitching.

### 2.16. qRT-PCR

The previously outlined standard protocol was employed to evaluate the expression of various cytokine and chemokine genes [16]. In brief, 800 µL of TRIzol (Invitrogen, Carlsbad, CA, USA) and 200 µL of chloroform were added to mouse tissues or a hamster cell pellet. These samples were subsequently lysed and centrifuged at 12,000 rpm for 5 min at 4 °C. RNA was then purified from the resulting aqueous phase using RNA easy mini columns (MNs), following the manufacturer’s instructions. To assess the quality of the RNA, it was subjected to electrophoresis on a formaldehyde gel to identify the 16s and 18s RNA bands, and it was also analyzed using a bioanalyzer. The quantity and purity of the RNA were measured with UV spectroscopy from Invitrogen at a 260/280 ratio. The PrimeScript 1st strand cDNA Synthesis Kit (Takara Bio, San Jose, CA, USA) was utilized as per the manufacturer’s instructions to synthesize the first-strand cDNA. The qRT-PCR procedure was performed using 96-well microtiter plates on a Bio-Rad system. The amplification process involved two steps with a reaction volume of 10 µL. The reaction mixture comprised 50 ng of cDNA, 10 pM of each primer listed in Supplementary Table S3, and SYBR green dye from Bio-Rad. Three replicates of each sample were analyzed, and the data were evaluated using the fold change (2^−ΔΔCt^) method. Changes in gene expression due to the treatment at different time points were compared to the control condition. The mRNA quantities of the analyzed genes were normalized using the corresponding reference housekeeping genes (β-actin or GAPDH). All primers used in the study were synthesized via IDT, and their sequences can be found in Supplementary Table S3.

### 2.17. Infection Experiments

The hamsters were subjected to an intraperitoneal challenge on day 35, two weeks after receiving a booster injection. This challenge involved exposing them to a 100-fold effective dose (ED50) of virulent *L. interrogans* serovar Pomona. The ED50 was determined using the methodology described in a previous study [27]. We monitored the hamsters for specific clinical signs throughout four weeks. These signs included hematuria, a loss of appetite, difficulties walking or breathing, disheveled fur, a hunched posture, prostration, or weight loss exceeding 20%. We observed these physiological indicators three times daily. Hamsters with severe clinical symptoms (reaching a moribund state) were euthanized after blood collection and recorded as deceased. For those hamsters that withstood the challenge, blood samples were taken, and they were sacrificed at the end of the monitoring period. We collected the kidneys, liver, and lungs under aseptic conditions to evaluate the bacterial load and histopathological changes.

### 2.18. Determination of Bacterial Burden

We assessed the bacterial quantity within the organs of all infected animals, including those that met the specified endpoint criteria and those that survived until the end of the experiment before being euthanized. To determine the bacterial load, we utilized the quantitative RT-PCR method, which was previously described [28]. The kidney, liver, and lung tissues were dissected into smaller fragments and preserved in RNAprotect (Qiagen) for subsequent procedures. We followed a standard protocol to extract the total DNA content from these tissues. Quantification was performed using the Bio-Rad Real-Time PCR System with the 2 × SYBR Green PCR Master Mix (Bio-Rad, Berkeley, CA, USA) and specific primers designed to amplify *Leptospiral* 16s rRNA and LipL32 genes. We employed 10-fold serial dilutions of bacterial DNA to establish a *Leptospiral* DNA standard curve, resulting in a range from 2 × 10^1^ to 2 × 10^9^ cells/mL.

### 2.19. Histopathology

The structural integrity of hamster tissues was preserved through collection, fixation, and immersion in a 10% neutral buffered formalin solution. Subsequently, these tissues were sliced into 5 μm sections using a microtome, stained with hematoxylin and eosin, and examined under light microscopy. An experienced veterinary pathologist, unaware of the sample identities, assessed the lesions induced by *Leptospira* in the infected organs. The severity of tubulointerstitial nephritis was determined using a grading system that ranged from 0 (indicating normal) to 3 (indicating severe) based on established criteria [28]. Likewise, the lung and liver pathology was assessed by counting the average number of inflammatory foci in 10X field of view. The grading scale for lung and liver pathology ranged from 0 (indicating normal) to 3 (indicating severe with more than 7 inflammatory foci).

### 2.20. Statistical Analysis

In most conducted experiments, the results were assessed using a one-way analysis of variance (ANOVA) along with the Dunnett hypothesis test unless stated otherwise. The data are presented as the average of three replicates, accompanied by the standard error of the mean (SEM). Statistical significance was determined using a significance threshold of *p* < 0.05.

## 3. Results

### 3.1. Leptospira Lipid A Is a Potent Adjuvant Capable of Inducing Strong Innate and Antigen-Specific Adaptive Immune Responses

**Figure 1:**
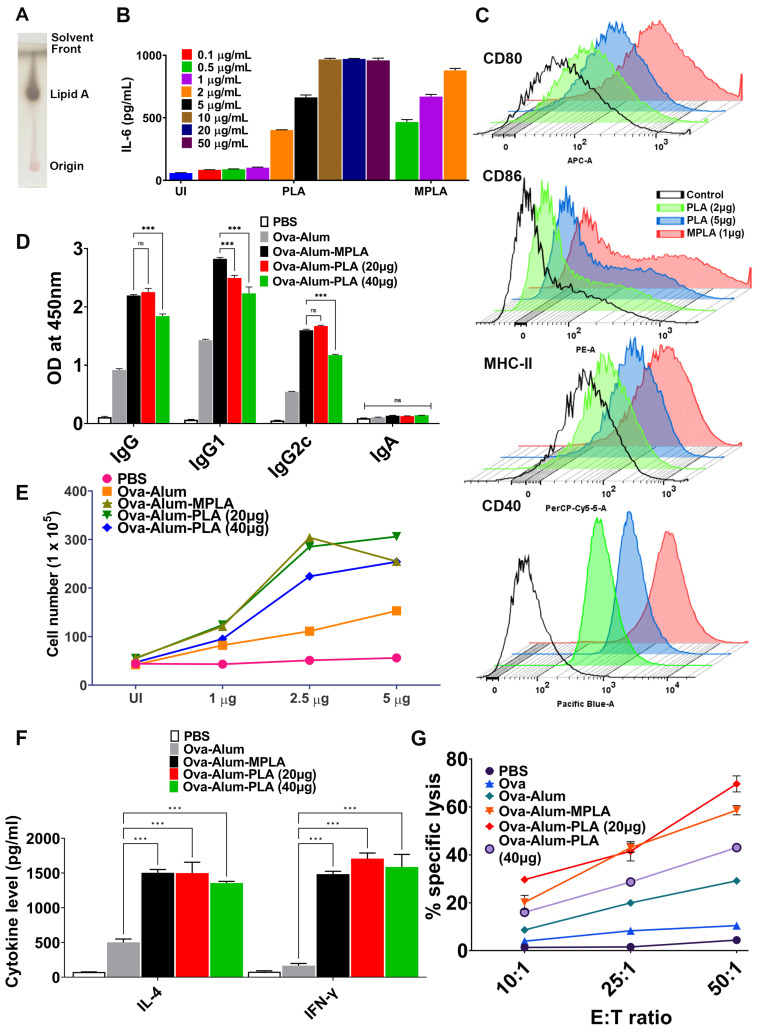
**Immunostimulatory and adjuvant activity of *Leptospira* lipid A (PLA).** (**A**) *Purification of PLA***:** PLA was purified, and bands were resolved and visualized through thin-layer chromatography (TLC) as mentioned in the Materials and Methods. (**B**) *Estimation of PLA-induced pro-inflammatory cytokines:* RAW264.7 cell lines were stimulated with MPLA (0.5–2 µg/mL) or PLA (0.1–50 μg/mL) for 24 h, and a culture supernatant was used to determine the IL-6 release via a sandwich ELISA. (**C**) *Analysis of PLA-induced expression of surface markers on mouse macrophages:* The RAW264.7 cells were treated with MPLA (1 µg/mL) or PLA (2 or 5 μg/mL) for 24 hrs, and the cells were harvested and stained with fluorochrome-conjugated antibodies and analyzed via flow cytometry. The control group indicates the uninduced samples. (**D**–**G**) *Adjuvant activity of PLA in vivo:* mice were immunized with OVA formulated in alum with or without PLA or MPLA, and antigen-specific humoral and cellular immune response was determined as described in Materials and Methods. (**D**) *Humoral immune response:* the OVA-specific antibodies (IgG, IgG1, IgG2c, IgA) in the serum (1:10,000 dilution) obtained on day 28 were determined via an ELISA, as explained in Material and Methods. (**E**) *Lymphocyte proliferation:* splenocytes obtained from different groups were stimulated with OVA (1 or 2.5, or 5 µg/mL), and cell proliferation was assessed after 72 hrs using the trypan blue size exclusion assay. (**F**) *Cytokine analysis:* the levels of IL-4 and IFN-γ in the culture supernatant of the stimulated splenocytes were analyzed via an ELISA. (**G**) *Analysis of cytotoxic T cells (CTLs):* The activity of CTLs obtained from various groups was determined using the LDH cytotoxicity kit as specified in the Materials and Methods Section. The one-way ANOVA was employed to calculate significant variations, with “***” denoting *p* ≤ 0.001 and “ns” indicating non-significance.

We purified lipid A from *Leptospira interrogans* serovars Pomona (PLA) using a standard protocol and analyzed it via thin-layer chromatography (Figure 1A). To assess its immunostimulatory activity, we stimulated mouse macrophages with varying doses of PLA and subsequently examined the production of pro-inflammatory cytokines, as well as the upregulation of costimulatory molecules (CD80, CD86, CD40), and a maturation marker (MHC-II). Our findings indicate that, similar to MPLA, PLA induces the activation of mouse macrophages, as evidenced by significant levels of IL-6 and the upregulation of CD80, CD86, CD40, and MHC-II. However, this effect was achieved at much higher doses, suggesting that PLA is less stimulatory than MPLA (Figure 1B,C). MPLA is known for its ability to enhance the antigen-specific immune response; we explored whether PLA has a similar effect when formulated with OVA and alum. The results demonstrated that OVA-alum-PLA induces significantly higher antibody levels than OVA-alum alone. However, these levels are similar to those induced by OVA-alum-MPLA (Figure 1D). Notably, OVA-alum-PLA leads to higher levels of both IgG1 and IgG2c antibodies but fails to induce mucosal antibody IgA (Figure 1D). To analyze the T cell response, we isolated splenocytes from different groups on day 28 and stimulated them with the recall antigen (OVA). The results showed that splenocytes isolated from OVA-alum-PLA group exhibited significantly higher levels of proliferation compared to the control group (LAV-alum) but similar to the levels induced by splenocytes from OVA-alum-MPLA (Figure 1E). Alum predominantly triggered a Th2 response, as evidenced by the production of mainly IL-4 and minimal levels of IFN-γ. However, both PLA and MPLA induced a mixed Th1/Th2 response characterized by significantly elevated levels of IL-4 and IFN-γ (Figure 1F).

Additionally, we investigated whether the OVA-alum-PLA formulation could induce cytotoxic T cells (CTLs) by assessing the lysis of target cells (DCs stimulated with OVA) using the LDH assay. The results indicated that CTLs obtained from animals immunized with either OVA-alum-PLA or OVA-alum-MPLA demonstrated a higher level of target cell lysis (70–80%) compared to OVA-alum (20–30%) at the highest E/T ratio (Figure 1G). It is important to note that effectors obtained from both OVA-alum-PLA and OVA-alum-MPLA did not exhibit lysis of non-specific targets (DCs activated with unrelated antigen, BSA), thus validating the specificity of CTLs. In summary, our results reveal that, similar to MPLA, PLA is a potent adjuvant capable of enhancing antigen-specific humoral and cellular immune responses.

### 3.2. PLA Enhances the Adaptive Immune Response against Leptospira Surface Antigens

**Figure 2:**
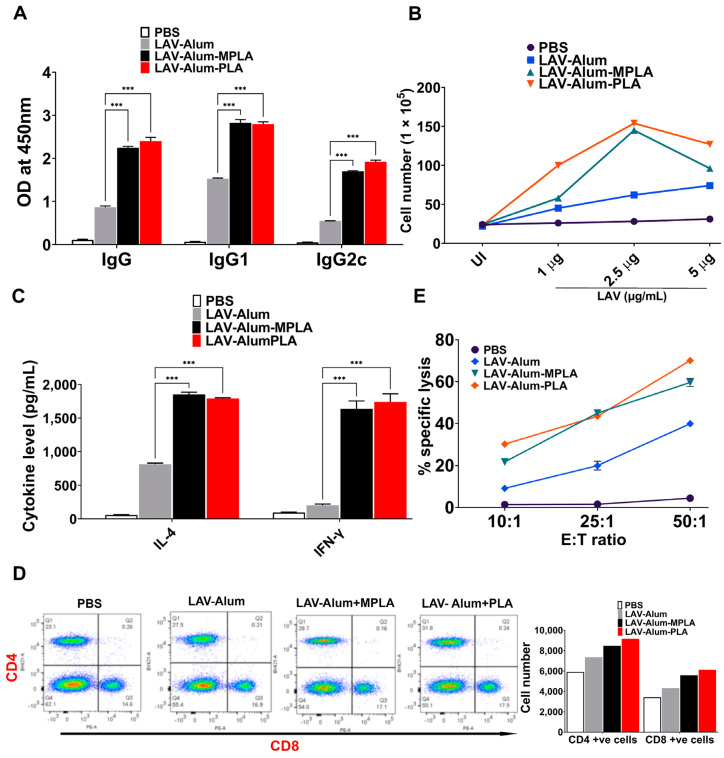
**PLA is a potent adjuvant that enhances the immune response against *Leptospira* surface antigen.** Mice were immunized with LAV formulated in alum with or without PLA or MPLA, and LAV-specific humoral and cellular immune response was determined as described in Materials and Methods. (**A**) *Evaluation of LAV-specific antibody response****:*** the level of LAV-specific antibodies (IgG, IgG1, and IgG2c) in the serum (1:10,000 dilution) obtained on day 28 was analyzed via an ELISA, as detailed in the Materials and Methods Section. (**B**) *Assessment of LAV-specific lymphocyte proliferation:* On day 28, splenocytes from the different immunized groups were isolated and stimulated with LAV for 72 hrs. Cell proliferation was evaluated using the trypan blue size exclusion assay. (**C**) *Analysis of cytokine production:* splenocytes were stimulated with LAV (5 µg/mL) for 48 hrs, and the release of IL-4 and IFN-γ cytokines in the culture supernatant was quantified using a sandwich ELISA kit following the manufacturer’s instructions. (**D**) *Examination of T cell phenotype:* splenocytes stimulated with LAV (2.5 µg/mL) for 24 h were stained with anti-mouse CD3, CD4, and CD8 and then analyzed using flow cytometry. (**E**) *CTL assay:* The activity of LAV-specific CTLs obtained from various groups was determined using the LDH cytotoxicity kit as specified in the Materials and Methods section. Significant differences were determined using one-way ANOVA. Statistical significance is denoted as “***” for *p* ≤ 0.001.

To assess the potential of PLA in bolstering the immune reaction to bacterial antigens, we tested its adjuvant activity with *Leptospira* immunoglobulin-like protein A (LigA), a surface protein expressed during infection and an established protective antigen. We employed a previously documented technique for cloning, expressing, and purifying the variable segment of LigA (LAV) in a soluble form [29]. Subsequently, we administered LAV to mice in combination with alum, alum-PLA, or alum-MPLA, and then evaluated the resulting humoral and cellular immune responses specific to LAV. Our findings suggest that, akin to OVA (Ovalbumin), PLA enhances the production of LAV-specific antibodies, including IgG and various isotypes, as well as the T cell response (Figure 2A,B). Specifically, LAV-alum-PLA prompts a balanced Th1/Th2 response, resulting in the production of both IgG2a/IFN-γ and IgG1/IL-4 (Figure 2C). Furthermore, both PLA- and MPLA-containing formulations amplified the generation of LAV-specific CD4+ and CD8+ T cells (Figure 2D). Moreover, both PLA and MPLA formulations produced LAV-specific cytotoxic T cells (CTLs). It is worth noting that these CTLs exhibit a higher level of target cell lysis, ranging from 50% to 70%, compared to CTLs derived from LAV-alum, which induce 20% to 30% lysis at the highest E/T ratio (Figure 2E). Additionally, effector cells derived from PLA- or MPLA-based formulations do not exhibit lysis of non-specific targets (such as dendritic cells stimulated with an unrelated antigen, OVA), thereby confirming the specificity of the CTL response. In summary, our findings underscore the capacity of PLA to augment the adaptive immune response against the *Leptospira* surface antigen.

### 3.3. PLA Mediates Its Adjuvant Effect via Cellular Recruitment and Activation at the Injection Site and Draining Lymph Nodes

**Figure 3:**
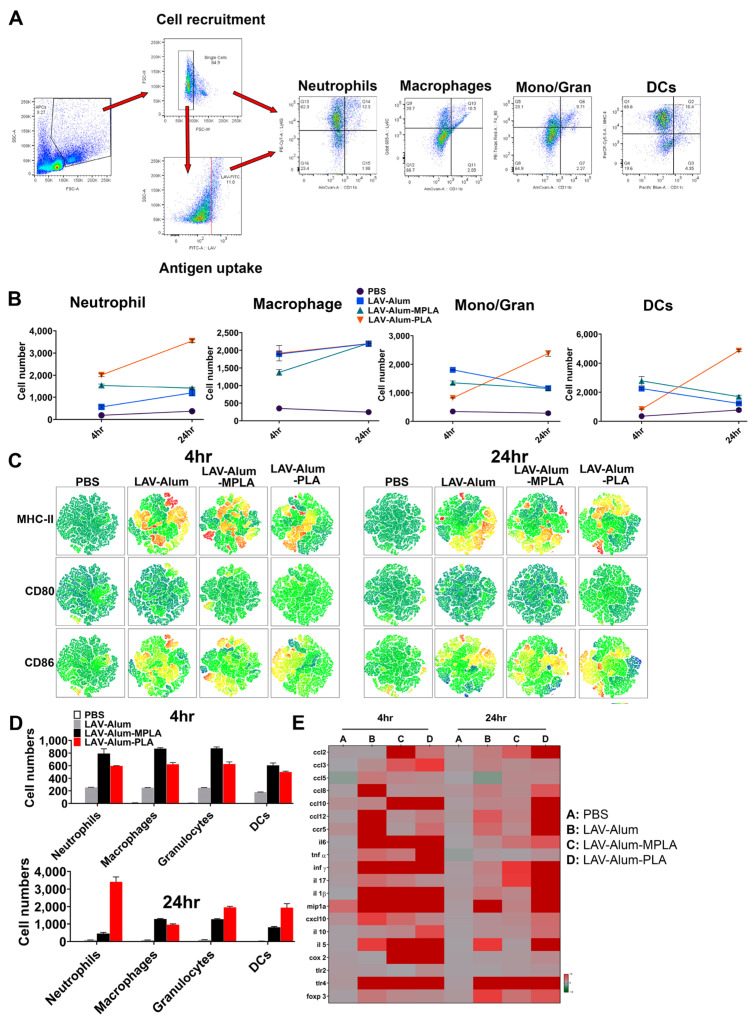
**PLA enhances the recruitment, activation, and antigen uptake by innate cells at the site of vaccine administration**. To evaluate PLA-induced recruitment, antigen uptake, and activation of innate immune cells, tissue from the injection site and DLNs were isolated at 4 h and 24 h for further analysis. (**A**) *Gating strategy:* the cells obtained from DLNs were stained with marker-specific fluorescent labelled antibodies as described in the methodology and then gated to identify the appropriate cell population. (**B**) *Analysis of cells recruited in DLNs:* the specific cell type recruited by each vaccine formulation at different time points was analyzed as detailed in the methodology. (**C**) *Activation status of cells recruited in DLNs:* The expression of surface markers (CD80, CD86, CD40, and MHC-II) was analyzed via flow cytometry and visualized in a tSNE plot. The heatmaps were generated to describe the intensity of specific markers; dark blue regions correspond to the lowest intensity of fluorescent antibody staining, while red indicates the highest intensity, with heatmap ranging from −1622 to 262856. (**D**) *Analysis of antigen uptake by APC:* the uptake of Alexa Fluor 488-labeled LAV by a different type of recruited APCs at 4 h and 24 h was analyzed via flow cytometry. (**E**) *Inflammatory responses at the injection site:* qRT-PCR analysis of cytokines, chemokines, and receptors at the injection site 4 and 24 h post-injection.

To understand how PLA functions as an adjuvant, we conducted an analysis of the local inflammatory response and the activation of innate immune cells at the site of vaccine administration and the draining lymph nodes (DLNs). This investigation was carried out at two different time points: 4 h and 24 h after injection. We used flow cytometry to examine pooled cells from the DLNs, assessing their numbers, types, and activation status (as shown in Figure 3A). Our findings revealed that, at the 4 h mark, PLA led to a significant recruitment of neutrophils (CD11b + Ly6G+). However, at 24 h post-injection, the recruitment of dendritic cells (DCs − CD11c + MCHII+) and granulocytes/monocytes (CD11b + Ly6C+) was considerably higher in the PLA group compared to MPLA and alum (as depicted in Figure 3B). On the other hand, MPLA-alum induced the early recruitment of neutrophils (CD11b + Ly6G+) and granulocytes/monocytes (CD11b + Ly6C+) (Figure 3B). Our tSNE analysis demonstrated that both PLA and MPLA significantly increased the expression of costimulatory molecules (CD80, CD86) and a maturation marker (MHCII) in the recruited immune cells (as shown in Figure 3C). Once we established that the PLA formulation generally resulted in robust cell migration and activation, we proceeded to analyze the uptake of the antigen (LAV labeled with Alexa FluorTM 488) by these cells. The flow cytometry analysis indicated that at 4 h, there was rapid detection and a significantly higher number of LAV+ cells in both the LAV-alum-PLA and LAV-alum-MPLA groups compared to LAV-alum (Figure 3D). Neutrophils and DCs predominantly took up the antigens in the PLA formulation, while macrophages did so in MPLA-immunized animals (Figure 3D). As adjuvants induce the expression of various cytokines and chemokines at the injection site to create a pro-inflammatory environment, we examined the PLA-induced expression of cytokines, chemokines, and their receptors using qRT-PCR. Our gene expression analysis at 4 and 24 h demonstrated that PLA modulated the expression of several cytokines/chemokines or their receptors (as illustrated in Figure 3E). While MPLA enhanced the expression of some of these cytokines and chemokines (ccl2, ccl5, ccl10, il-1b, mip1a, and cxcl10) at an early time point (4 h), PLA enhanced their expression at both 4 and 24 h. A mock injection with PBS induced a baseline expression of these cytokines/chemokines due to needle-induced injury (Figure 3E).

### 3.4. PLA-Formulated Vaccine Induced Sustained Response with the Generation of Immunological Memory

**Figure 4:**
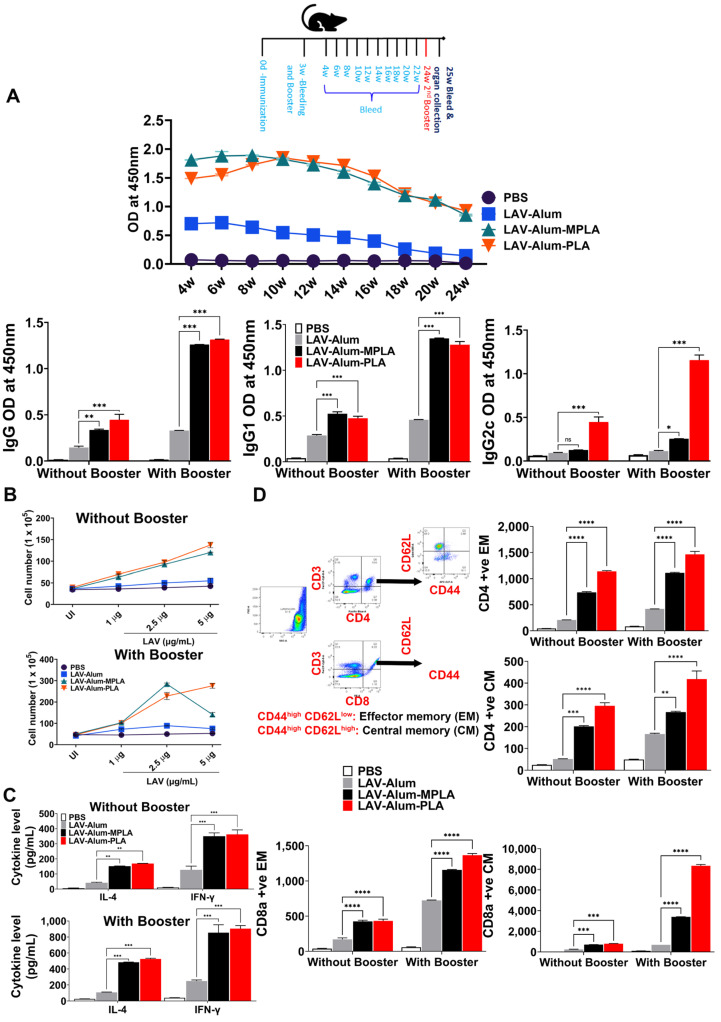
**Determination of the long-term immune memory induced by PLA-based vaccine formulation.** The animals immunized with PBS, LAV-alum, LAV-alum-MPLA, or LAV-alum-PLA and boosted on the 21st day were bled every week for 24 weeks. The animals were euthanized or given additional antigen boosters (without adjuvant), and spleens were collected after one week to determine memory T cells. (**A**) *The humoral (antibody) memory response:* the levels of LAV-specific IgG at various time points or isotypes (IgG1 or IgG2c) in the serum were analyzed via an ELISA as described in the Materials and Methods. (**B**) *The T cell memory response:* splenocytes isolated from mice euthanized at 24 w and 25 w were stimulated with LAV for 72 h; cell proliferation was assessed via trypan blue size exclusion assay. (**C**) *Estimation of cytokine levels:* 48 hrs after splenocyte stimulation with antigens (LAV; 5 µg/mL), using ELISA, the IL-4 and IFN-γ cytokines in culture supernatant as per the manufacturer’s instructions. (**D**) *Analysis of memory phenotype:* The splenocytes were stained with fluorescent-labeled antibodies specific against CD3, CD4, CD8, CD62L, and CD44, and we then performed flow cytometry analysis. Representative flow cytometry plots demonstrated the gating strategy for analyzing central (CD44^high^ and CD62L^high^) and effector (CD44^high^, CD62L^low^) memory response in CD4 and CD8 T cell populations. The numbers of positive cells expressing CD44 and CD62L over the total of CD4+/CD8+ T cells were plotted as histograms. The significant differences were calculated using one-way or two-way ANOVA, with the symbols ****, ***, **, *, and ns indicating *p* ≤ 0.0001, *p* ≤ 0.001, *p* ≤ 0.001, *p* ≤ 0.05, and non-significant, respectively.

**Figure 5:**
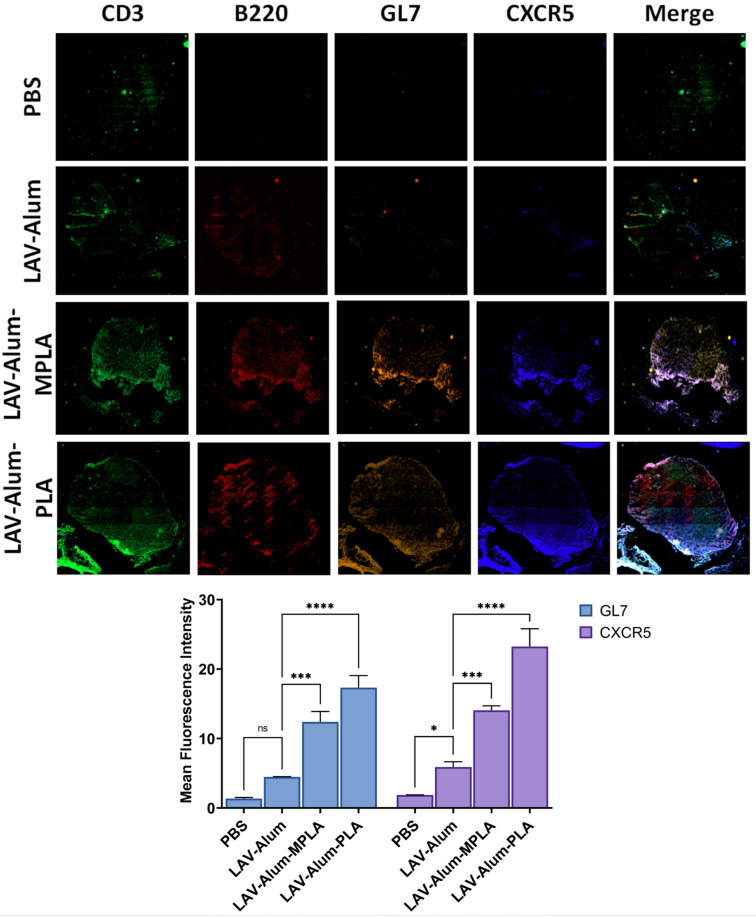
**Formation of Germinal Centers (GCs) in the draining lymph nodes.** The formation of GCs in DLNs of various groups was assessed on the 28th day post-immunization. Frozen tissue sections of DLNs were processed and later stained with fluorochrome-conjugated anti-mouse CD3/CD45R/B220/GL-7/CXCR5 antibodies as described in the methodology. The sections were mounted and examined under a fluorescent microscope using tile scanning and then processed using the ZEN software version 3.7 (stitching tool) as described in the methodology section. Data represent three different experiments. Significant differences were calculated using the one-way ANOVA (****, ***, *, and ns indicate *p* ≤ 0.0001, *p* ≤ 0.001, *p* ≤ 0.05, and non-significant, respectively).

After observing that the PLA-formulated vaccine stimulated a strong initial immune response, we were keen to investigate its ability to establish a long-lasting memory response, a critical factor for an effective vaccine. We analyzed the antibody levels in various groups for up to 24 weeks (approximately 6 months) following immunization to evaluate the long-term memory response. Both the LAV-alum-PLA and LAV-alum-MPLA formulations exhibited a sustained humoral response, as indicated by detectable antibody levels even at the 24-week mark after immunization (Figure 4A). In contrast, the LAV-alum group showed significantly lower antibody levels with a subsequent decline in titers at the 24-week time point. While both LAV-alum-PLA and LAV-alum-MPLA induced high levels of IgG and IgG1 antibodies, only LAV-alum-PLA triggered a noteworthy increase in IgG2c antibody levels (Figure 4A). To assess the development of immunological memory with these adjuvants, we administered an antigen booster without an adjuvant and observed an enhanced antibody response in both the LAV-alum-PLA and LAV-alum-MPLA groups (Figure 4A). In order to evaluate the memory T cell population, we stimulated the splenocytes with a recall antigen (LAV). Our results indicated significantly higher lymphocyte proliferation and cytokine production (IL-4, IFN-gamma) in both the LAV-alum-PLA and LAV-alum-MPLA groups (see Figure 4B,C). Similarly, lymphocytes obtained after the booster displayed significantly increased proliferation and cytokine production (Figure 4B,C). We then examined the central memory (CD44high and CD62Lhigh; TCM) and effector memory (CD44high and CD62Llow; TEM) phenotype of CD4 and CD8 T cells. Animals that were immunized with LAV-alum-PLA or LAV-alum-MPLA exhibited significantly higher levels of both central and effector memory CD4 and CD8 T cells compared to those immunized with LAV-alum (Figure 4D). The generation of long-lived plasma cells known for secreting high-affinity antibodies heavily depends on the formation of Germinal Centers (GCs), which are also crucial for the development of immune memory. To determine if the elevated and long-lasting antibody response induced by LAV-alum-PLA is associated with its influence on GCs, we examined inguinal lymph nodes collected from euthanized animals 28 days post-immunization. These lymph nodes were sectioned and stained with fluorescent antibodies targeting B220 (a B-cell marker), CD3 (a T cell marker), GL7 (a B-cell GC marker), and CXCR5 (GC-resident T follicular helper cell marker). An immunofluorescence analysis revealed a higher frequency of B220 + GL7+ and CD3 + CXCR5+ cells in mice immunized with either LAV-alum-PLA or LAV-alum-MPLA, indicating an increased number of lymph node GCs compared to those immunized with LAV-alum (Figure 5).

### 3.5. PLA-Formulated Vaccine-Induced Sterile Immunity against the Disease in a Hamster Model

**Figure 6:**
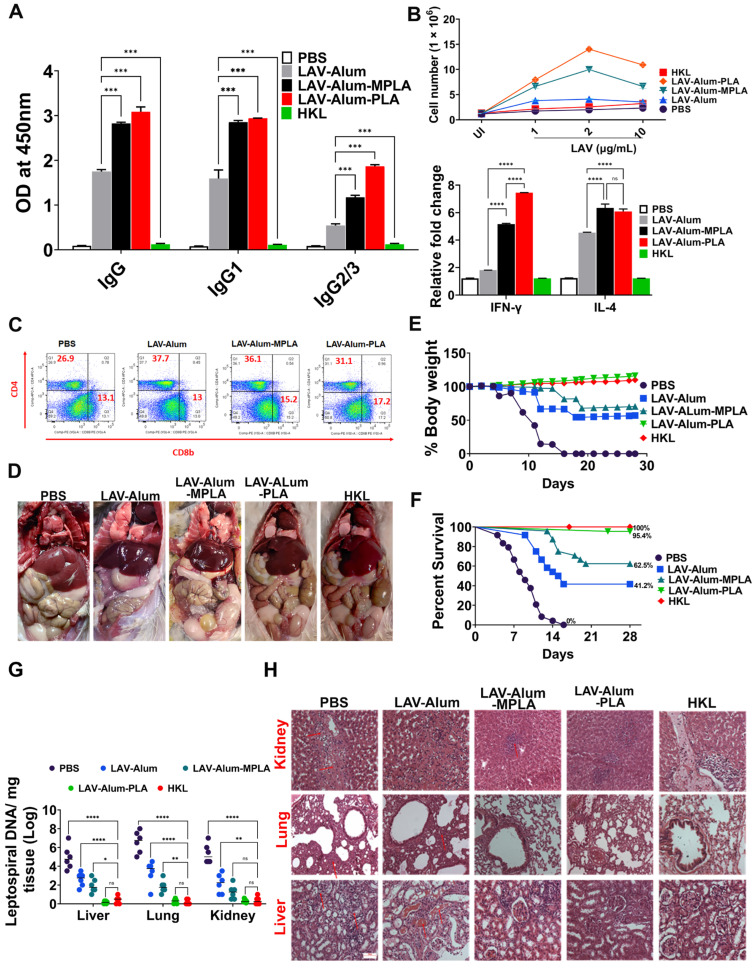
**PLA enhances the immune response and protective efficacy in the hamster leptospirosis model.** Immune response and protective efficacy of various vaccine formulations were assessed in the hamster model as detailed in the methodology. (**A***) Serum antibodies:* LAV-specific total IgG and subtype (IgG1 and IgG2/3) antibody levels were measured in immunized animal serum (1:10,000 dilution) on the 35th day using ELISA. (**B**) *Cell proliferation and cytokine response:* The splenocytes obtained from various groups were stimulated with LAV, and cell proliferation was analyzed using trypan blue size exclusion after 48–72 h. Stimulated cells were harvested at 24 h, and IL-4 and IFN-γ cytokine expression was analyzed using qRT-PCR. (**C**) *T cell analysis:* the flow cytometry identified LAV-specific T cell phenotypes by gating on a selection of CD4+ CD8b+ T cells. (**D**) *Clinical manifestations:* the clinical pathology was assessed by examining the gross appearance of the organs of the animals that either died or were euthanized according to the predetermined endpoint criteria. (**E**) *Bodyweight:* The animals challenged on day 35 were monitored for any changes in body weight until day 63. Individual body weights are shown as a percentage compared with the pre-challenge weight. (**F**) *Survival analysis:* the survival rate of the animals was monitored until 28 days post-challenge and was analyzed using Kaplan–Meier’s plot based on criteria described in the methodology. (**G**) *Leptospira colonization in the organs:* bacterial load per gram of tissue (lung, liver, and kidney) was determined via RT-PCR, quantifying the LipL32/16s gene. (**H**) *Histopathological analysis:* Representative images of hematoxylin and eosin-stained tissues (lung, liver, and kidney) were obtained from various groups of animals that died or survived after the challenge (scale bars, 10 μm). Significant differences were determined using either one-way or two-way ANOVA. The symbols ****, ***, **, *, and ns represent *p*-values of ≤0.0001, ≤0.001, ≤0.001, ≤0.05, and non-significant, respectively.

We then evaluated the effectiveness of a vaccine formulated with PLA in generating a robust immune response and its relationship with disease protection in a hamster model. We compared the performance of the PLA-formulated vaccine with the standard killed vaccine (HKL). Our analysis of the antibody response on day 35, which was conducted two weeks after the booster, revealed significantly higher levels of IgG antibodies in animals vaccinated with both the LAV-alum-PLA and LAV-alum-MPLA formulations when compared to those receiving LAV-alum alone. An analysis of the IgG isotypes generated by both the PLA and MPLA formulations revealed that there was preponderance of IgG1 with significant levels of IgG2/3 (as shown in Figure 5). Additionally, apart from the generation of antibodies against LAV, a significant amount of antibodies was also produced against PLA in animals immunized with LAV-alum-PLA (Supplementary Figure S1). Furthermore, when we isolated lymphocytes from the LAV-alum-PLA or LAV-alum-MPLA vaccinated groups, we observed significantly higher levels of lymphocyte proliferation and an increased expression of IL-4 and IFN-γ transcripts compared to the LAV-alum group (as depicted in Figure 6B). In contrast, the animals immunized with HKL did not exhibit a significant humoral or cell-mediated response specific to the LAV antigen (Figure 6A,B). Our T cell analysis using flow cytometry revealed a notable increase in CD4+ cells in all the vaccinated groups, except for the PBS control group. However, the enhancement of CD8 T cells was observed only in the groups that received the PLA- and MPLA-based formulations (Figure 6C). To assess the correlation between the immune response and protection, we subjected the vaccinated animals to a challenge with virulent *Leptospira* on the 35th day and evaluated their protective efficacy based on several parameters including progressive weight loss, survival rates, histopathological findings, and bacterial load. As previously reported in other studies, we considered a ≥20% weight loss in animals as the endpoint criterion to prevent spontaneous death. The control group receiving PBS displayed necrosis and small areas of gross and microscopic pulmonary hemorrhage, which are typical signs of acute leptospirosis (Figure 6D). Conversely, the LAV-alum and LAV-alum-MPLA groups exhibited milder disease features. In contrast, the LAV-alum-PLA and HKL groups appeared to be close to normal (Figure 6D). The PBS group experienced progressive weight loss, with animals unable to survive beyond 12 days post-challenge (as depicted in Figure 6E,F). The LAV-alum and LAV-alum-MPLA groups initially showed progressive weight loss but began to regain weight after 18 days post-challenge, with average survival rates of 50% and 67%, respectively as determined from four independent experiments. In contrast, the LAV-alum-PLA and HKL groups did not experience a decrease in body weight, with average survival rates of 95.4% and 100% respectively (Figure 6E,F and Supplementary Table S1). We also measured the bacterial load in the infected organs by quantifying the DNA copy number per milligram of tissue using qRT-PCR. The results indicated that the bacterial load in the liver, lung, and kidney of the PBS group was significantly higher (*p* > 0.0001) than in the other vaccinated groups (Figure 6G). The animals immunized with LAV formulated with alum or alum-MPLA had a significantly lower bacterial load than the control group. However, they exhibited a significantly higher bacterial load in the lungs (*p* > 0.01) and liver (*p* > 0.05), with no significant difference in the kidney when compared to the HKL-immunized animals. Notably, the LAV-alum-PLA group showed a similar or even lower bacterial load than the HKL group, and in some animals, the bacterial load in their organs was below the detection limit (Figure 6G).

Histopathological analysis is a sensitive method for detecting sublethal *Leptospiral* infection and is an essential parameter for assessing vaccine efficacy. Examinations of the liver, lung, and kidney revealed varying degrees of lesions, necrosis, and cell infiltration (as shown in Figure 6H). The PBS control animals exhibited severe lesions in the kidneys, characterized by marked chronic tubulointerstitial nephritis, severe atrophy, fibrosis, and lymphocyte infiltration. The liver showed centrilobular necrosis with numerous inflammatory foci, while the lungs displayed edema and foci with intense hemorrhages (as depicted in Figure 6H). The pathological scoring of the organs from these vaccine groups indicated that 80% of the animals were normal in the HKL group, with 20% showing mild lesions. In the LAV-alum-PLA group, 85–90% of animals were normal, while 10–15% had mild lesions. The LAV-alum-MPLA group had 40% of animals classified as normal, with 35% having moderate and 25% having mild lesions. In contrast, in the LAV-alum group, all the animals showed lesions, with 20% having mild, 45% having moderate, and around 35% having severe lesions (Figure 6H and Supplementary Table S2).

## 4. Discussion

Currently, the primary approach for treating and preventing leptospirosis during an outbreak relies on broad-spectrum antibiotics for therapeutic and prophylactic purposes [30]. However, indiscriminate antibiotic use to protect susceptible humans and animals from infection and subsequent transmission after a leptospirosis outbreak is impractical and can potentially lead to antibiotic resistance [31]. Although vaccination is the most effective and cost-efficient intervention, the existing killed vaccine offers only short-term and serovar-specific protection, falling short of complete immunity [3]. This vaccine is primarily administered to animals, and due to its toxicity, it is avoided in humans [2]. To address the limitations of conventional whole-cell vaccines, efforts have been made to identify protective surface antigens for developing modern subunit vaccines [6,32]. Nonetheless, these vaccines require potent adjuvants to enhance their efficacy [33]. Bacterial LPS, including lipid A, has been extensively studied as an immunostimulatory agent, with numerous investigations exploring the structure–activity relationship of these Toll-like receptor 4 (TLR4) ligands and their potential as adjuvants [34]. Since the clinical approval of monophosphoryl lipid A (MPLA) as an adjuvant, particularly in the AS0X series by GSK, its use has expanded as a crucial component in vaccines against various infectious diseases [35,36,37]. The development of synthetic Glucopyranosyl lipid A (GLA) further underscores the significance of bacterial lipid A as an adjuvant [38]. Our recent study demonstrated that MPLA can enhance the antigen-specific immune response and provide protection against *Leptospira* infection in a hamster model [16]. Given that *Leptospira* LPS is a major surface antigen, the protective immunity generated by whole-cell vaccines is believed to be primarily mediated by anti-LPS IgMs [39]. Indeed, several studies have shown the role of LPS in conferring protection against challenges in animal models [40]. This is further supported by the fact that TLR4 (which LPS binds as a ligand) plays a pivotal role in protecting against the disease [23]. *Leptospira* LPS is naturally less endotoxic and atypical as it activates both TLR2 and TLR4 to induce an inflammatory response [24,41]. Moreover, there is no need for structural modifications to LPS or lipid A to minimize toxicity while preserving the adjuvant effect. Considering these findings, we were motivated to assess the adjuvant potential of *Leptospira* lipid A.

We isolated LPS and lipid A from three significant pathogenic serovars (Icterohaemorrhagiae, Hardjo, Pomona) of *Leptospira interrogans* that infect various hosts and examined their innate activity on mouse macrophages. Regardless of the serovar from which it is purified, all lipid A molecules induced a pro-inflammatory response through the TLR4 MyD88-dependent pathway, leading to macrophage activation. However, it is worth noting that the effective dose required for this response was considerably higher than that of MPLA (unpublished data). Interestingly, LPS isolated from serovar Pomona appeared to activate macrophages through a less inflammatory TRIF pathway, resembling the activity of MPLA [20]. These findings prompted us to explore the potency of PLA in enhancing antigen-specific immune responses. Our in vitro results suggest that PLA may be less stimulatory than MPLA, as it requires a much higher dose to induce pro-inflammatory cytokines and the expression of activation and maturation markers, as shown in Figure 1B. This difference in activity could be attributed to the source of these molecules, with MPLA being a highly pure commercial product, while PLA is lab-purified. Additionally, the variation in immunostimulatory activity between MPLA and PLA might also arise from structural distinctions in their respective lipid A molecules. Although the lipid A structure appears to be conserved within the three pathogenic serovars we investigated (as indicated by our unpublished data), recent reports suggest structural diversity in lipid A across the broader genus of *Leptospira* [42].

Our in vivo data indicate that alum-PLA elicits significantly higher levels of OVA and LAV-specific humoral and cellular immune responses compared to those induced by alum alone, albeit at a much higher dose (20 µg) than MPLA (5 µg), confirming the generally lower stimulatory activity of PLA compared to MPLA (as shown in Figure 1 and Figure 2). The mechanism through which PLA enhances antigen-specific humoral and cellular immune responses appears to be similar to MPLA and is primarily mediated by an enhanced recruitment, activation, maturation, and uptake of antigens by antigen-presenting cells (APCs), ultimately leading to the activation of B and T cell responses [22]. Our results support this, demonstrating the rapid appearance of antigen-loaded and activated APCs in the draining lymph nodes for both PLA- and MPLA-formulated vaccines (as shown in Figure 3B,D). The cytokines induced at the injection site by the PLA-formulated vaccines correlate with the cellular events detected in the draining lymph nodes (Figure 3E) [43]. Chemokines such as CCL2 and CCL3, enhanced by both the PLA and MPLA formulations, are known to promote the recruitment of monocytes and immature DCs [44]. Differentiated APCs play a critical role in inducing adaptive responses, as they can take up antigens and migrate to the draining lymph nodes [44]. Our results demonstrate that the formulation of PLA with alum has resulted in optimal APC recruitment and activation in the draining lymph nodes. This may be partly attributed to alum’s ability to prolong the cytokine response to PLA. The direct activation of APCs, such as DCs, by PLA or MPLA may also be crucial for sustaining an antibody response. In fact, it is believed that the direct activation of DCs by TLR agonists, rather than cytokines alone, can enhance their ability to promote antigen-specific immune responses [45]. The immunostimulatory activity of PLA or MPLA may indirectly play a significant role in enhancing the antibody response, as pro-inflammatory cytokines are known to stimulate T helper cells specialized in assisting B-cells [46]. Although we have not tested the direct effect of PLA on T cells, it is possible that PLA could activate these cells through TLR4 and enhance the expression of activation markers (CD69) and costimulatory ligands (CD40L) [47].

The strong and similar levels of innate and antigen-specific adaptive immune responses generated by both MPLA and PLA can be attributed, in part, to the structural similarities and differences between these two immunostimulatory molecules. Both lipid A molecules contain amide linkages, with PLA having a higher number of them. These amide linkages may lead to a more stable and robust interaction with TLR4, resulting in a strong innate immune response followed by a subsequent adaptive immune response [48,49]. This phenomenon has been previously observed during *N. meningitidis* infection, where the robust immune response is attributed to the strong interaction of its lipid A (containing amide-linked fatty acids) with TLR4 [50]. Our results have demonstrated that both MPLA- and PLA-based vaccine formulations induced long-term and persistent immune responses, ultimately leading to the establishment of immunological memory. This persistence can also be attributed to the presence of amide linkages in their lipid A structures (as shown in Figure 4). Amide linkages contribute to stability and resistance to hydrolysis by acylase enzymes, allowing lipid A to persist for a longer duration and sustain the immune response. Another structural difference between PLA and MPLA is that PLA contains two unsaturated fatty acyl chains. Unsaturated fatty acids within the lipid A structure have been shown to enhance TLR recognition and influence the host’s immune response [51]. These unsaturated fatty acids can induce structural modifications in lipid A, such as the formation of kinks, which facilitate a better fit into the hydrophobic pocket of MD-2, an accessory protein required for TLR4 signaling. This enhanced binding affinity leads to the increased activation of TLR4-mediated immune responses. In *Salmonella enterica* serovar Minnesota, the modification of lipid A with unsaturated fatty acids has been associated with increased cytokine production and inflammation in host cells [52]. Molecular docking studies have further confirmed that the kinks in lipid A from *E. coli*, caused by unsaturated fatty acids, result in more extensive interactions with TLR4 and MD-2 compared to lipid A with saturated fatty acids [53]. Despite containing more amide linkages and unsaturation, PLA exhibits lower stimulatory activity than MPLA. This difference in activity might be attributed to the presence of methylated phosphate groups in PLA, which could induce distinct recognition patterns with TLR4 and TLR2 [54]. However, the amide linkages in PLA contribute to its stability, allowing it to induce a sustained but lower level of stimulatory activity.

To evaluate the effectiveness of the LAV-alum-PLA formulation in conferring protection against disease, we conducted experiments using a hamster model of leptospirosis. Our findings demonstrate that hamsters immunized with LAV-alum-PLA exhibited significantly elevated levels of antigen-specific antibodies and increased concentrations of both IL-4 and IFN-gamma compared to those immunized with LAV-alum (refer to Figure 6A,B). This robust immune response was associated with protection, as indicated by many surviving animals with reduced bacterial burden and fewer lesions in their vital organs (see Figure 6D–F). Interestingly, while LAV-alum-PLA and LAV-alum-MPLA induced similar immune responses, the former provided superior protection, akin to the levels observed with HKL, in terms of the number of survivors and the reduction in bacterial load in organs (see Figure 6G). This observed difference in the level of immune response and protection between LAV-alum-MPLA and LAV-alum is consistent with prior research, including our studies, where MPLA-based formulations conferred significantly higher levels of protection (ranging from 60 to 67%) compared to alum [12,13,16]. It is worth noting that PLA induced better protection than the MPLA-based formulation and, in some cases, even achieved sterilizing immunity despite generating similar levels of immune response specific to LAV. We hypothesized that, aside from anti-LAV antibodies, anti-PLA antibodies may enhance protection [55]. Indeed, we detected significant levels of anti-PLA antibodies in animals immunized with LAV-alum-PLA (see Supplementary Figure S1). The improved efficacy of the formulation may also be attributed to the activation of additional innate pathways, particularly those involving signaling through TLR2; however, further testing and validation of this hypothesis are required [56]. It is essential to highlight that the protection provided by HKL is achieved by generating an immune response against a range of surface antigens, including LPS. Notably, LAV-specific antibodies and T cells did not contribute to protection, as the bacteria cultured in vitro do not express LigA [57]. Considering the variation in O-antigens among different serovars and the conservation of lipid A among pathogenic species, it is plausible that the PLA-formulated vaccine may induce cross-protection against challenge with heterologous serovars. However, this remains an active area of investigation, and ongoing experiments are addressing this aspect.

## 5. Conclusions

**Figure 7:**
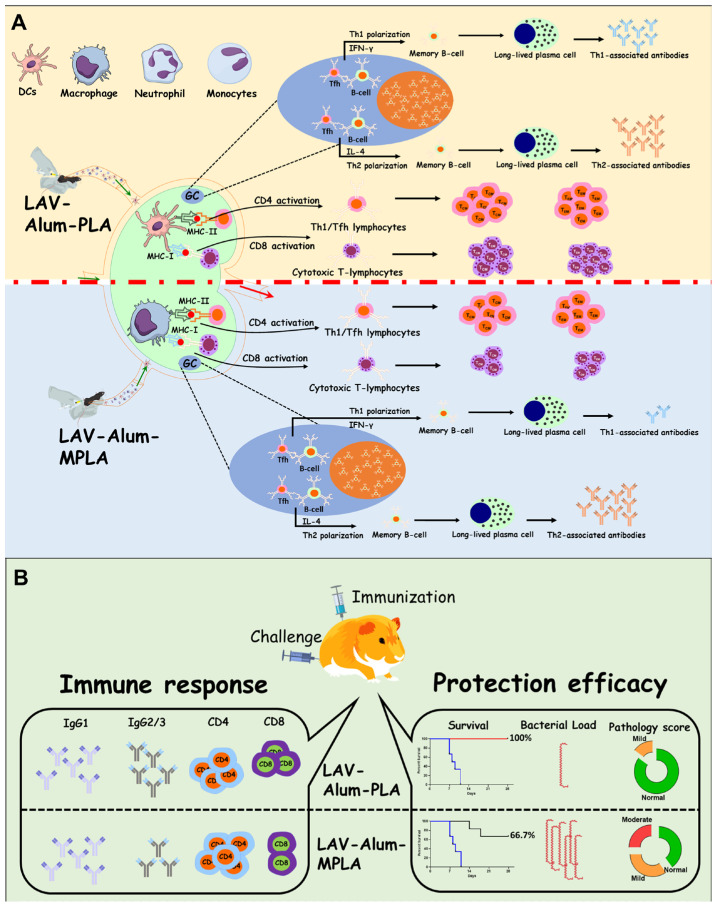
**Schematic representation depicting the difference in immune response and protective efficacy induced by PLA- or MPLA-based vaccine formulation.** (**A**) *Immune response in mice*: Both formulations increased the recruitment and activation of innate immune cells at the sight of injection and draining lymph nodes. While PLA-based formulation increased the recruitment and activation of dendritic cells and neutrophils, MPLA promoted the activation of monocytes. The formulations induced mixed Th1/Th2 response by generating IgG1/IL-4 and IgG2c/ IFN-γ. While both formulations generated long-term memory response, PLA induced significantly higher levels of GC reaction correlating to higher levels of Th1-associated antibodies. It also induced higher levels of central and effector memory T cells. (**B**) *Immune response and protective efficacy in a Hamster model*: Both the formulations induced strong antibody responses with significant levels of IgG1, but PLA induced significantly higher levels of IgG2/3. PLA induced more CD4 and CD8T cells, generating higher levels of IFN-g. The immune response correlated to enhanced protective efficacy, as the PLA-based formulation had more survivors with reduced or undetectable bacterial load and mild or no lesions in vital organs.

Our study has provided evidence that *Leptospira* lipid A is a potent adjuvant, activating the innate immune system and enhancing a sustained, antigen-specific protective immune response. Interestingly, the PLA-formulated vaccine demonstrated better efficacy when compared to the vaccine formulated with MPLA. Notably, it showed effectiveness equivalent to the standard killed vaccine, as depicted in Figure 7. However, it is essential to note that the killed vaccine is known to have toxic properties and is unsuitable for use in humans. In contrast, the PLA-formulated vaccine is a promising alternative due to its lower inherent endotoxicity. Furthermore, our experiments in mice and hamsters did not reveal any signs of local or systemic toxicity associated with the PLA-based vaccine.

Lipid A is an important protective antigen; however, without the help of T cells, it induces a short-term immune response and fails to generate memory. Since physically mixing PLA with protein antigens could enhance antigen-specific immune responses, conjugating PLA with LAV would be a better strategy, as antigen co-delivery and the subsequent co-activation of APCs could lead to the generation of a robust immune response simultaneously against both LAV and PLA. Hence, future efforts should be directed towards conjugating LAV with synthetic PLA to develop a conjugate vaccine against this important zoonosis.

## Data Availability

All the data provided in the manuscript are available upon request from corresponding author.

## Supplementary Materials

The following supporting information can be downloaded at https://www.mdpi.com/article/10.3390/vaccines11121824/s1: Supplementary Figure S1. Generation of anti-PLA antibodies in hamsters immunized with LAV-alum-PLA. Table S1: The survival data of immunized animals following infection with a virulent strain of *Leptospira* for 28 days. Table S2: Histopathological scores of different organs in hamsters following infection with virulent *Leptospira*. Table S3: The primers used for qRT-PCR analysis in the study.
