# Supplementary material for: Leptospira Lipid A Is a Potent Adjuvant That Induces Sterilizing Immunity against Leptospirosis
Source: Vaccines (Basel). 2023 Dec 6;11(12):1824. doi: 10.3390/vaccines11121824 (PMC10748165; doi:10.3390/vaccines11121824)
Supplement: Supplementary file 1 [file vaccines-11-01824-s001.zip › vaccines-2531990-supplementary.pdf]

Supplementary data

Leptospira Lipid A is a potent adjuvant that induces sterilizing immunity against Leptospirosis.

Vivek Varma et. al.

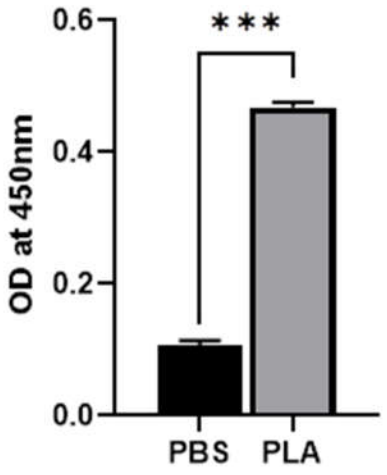

Figure S1. Generation of anti-PLA antibodies in hamsters immunized with LAV-Alum-PLA. PLA-specific total IgG antibody levels in the serum (1:10,000 dilution) of the immunized animals collected on the 35th day were measured by ELISA. The symbol *** represent p-value ≤0.001.

Supplementary tables

Table. S1: The survival data of immunized animals following infection with a virulent strain of Leptospira for 28 days

Group	Survival			
	Experiment 1	Experiment 2	Experiment 3	Experiment 4
PBS	0/6	0/6	0/6	0/6
LAV-Alum	2/6	4/6	3/6	3/6
LAV-MPLA-Alum	4/6	3/6	4/6	4/6
LAV-PLA-Alum	4/4	5/6	6/6	6/6
HKL	6/6	6/6	5/6	6/6

Table. S2: Histopathological scores of different organs in hamsters following infection with virulent *Leptospira*

Group	Pathology score											
	Experiment 1			Experiment 2			Experiment 3			Experiment 4		
	**Liver**	**Lung**	**Kidney**	**Liver**	**Lung**	**Kidney**	**Liver**	**Lung**	**Kidney**	**Liver**	**Lung**	**Kidney**
PBS	3,2,2,3,1,2	3,2,2,3,1,2	3,3,2,3,3,3	3,2,3,3,2,3	3,2,2,3,3,2	3,3,1,2,3,2	1,3,3,2,2,3	3,3,2,3,2,3	3,2,3,2,2,3	3,3,2,3,2,2	3,3,2,2,2,3	3,2,3,3,2,3
LAV-Alum	2,1,3,1,2,1	2,1,3,1,2,1	2,2,3,2,3,2	1,2,3,2,2,3	1,2,2,3,1,2	1,1,2,2,3,1	2,1,3,2,2,2	2,2,3,3,2,1	3,2,2,1,2,3	3,1,2,3,2,2	3,1,2,1,2,3	1,2,3,1,2,2
LAV-MPLA-Alum	1,0,1,0,2,0	1,0,1,0,2,0	0,1,0,2,2,1	1,1,0,1,2,1	1,1,0,0,2,1	1,2,1,1,2,1	2,1,1,1,2,2	2,1,1,2,2,1	1,1,2,1,2,2	1,2,2,1,2,1	2,1,1,1,2,2	1,1,2,2,2,1
LAV-PLA-Alum	1,0,1,0	0,0,1,0	0,1,1,0	1,0,0,1,1,2	0,1,0,0,0,1	0,1,0,1,0,0	0,1,0,1,0,0	0,1,1,0,0,0	0,0,0,1,0,0	0,1,1,0,0,0	0,1,0,1,1,0	0,1,1,0,0,0
HKL	0,0,0,0,1,0	0,0,0,0,1,0	0,1,0,0,0,1	0,0,1,0,1,0	0,0,1,1,0,0	1,0,0,0,0,0	0,1,0,0,1,0	0,0,0,1,0,0	0,1,1,0,0,1	0,1,1,0,0,0	1,0,0,0,1,0	0,1,0,1,0,0

Scores- 3 - Severe lesions
2 - Moderate lesions
1 - Mild lesions
0 - Normal

Table. S3: Primers used for qRT-PCR analysis in the study

Gene name	**Primer Sequence (5'→3')**	
Beta-actin	**F**	CACCCACACTGTGCCCATCTACGA
	**R**	GGATGCCACAGGATTCCATACCCA
ccl2	**F**	ACGTGTTGGCTCAGCCAGA
	**R**	ACTACAGCTTCCTTTGGGACACC
ccl3	**F**	ACTGCCTGCTGCTTCTCCTACA
	**R**	AGGAAAATGACACCTGGCTGG
ccl5	**F**	AGATCTCTGCAGCTGCCCTCA
	**R**	GGAGCACTTGCTGCTGGTGTAG
ccl8	**F**	CTTTGCCTGCTGCTCATAG
	**R**	GCACTGGATATTGTTGATTCTC
ccl10	**F**	TACTGCTGGCTCACCTC
	**R**	ATCTGTCTTGTGAAACCC
ccl12	**F**	GCTACCACCATCAGTCCTC
	**R**	CTGGCTGCTTGTGATTCTC
ccr5	**F**	ACACTCAGTATCATTTCTGG
	**R**	GGATCAGGCTCAAGATGACC
Il6	**F**	TGGAGTCACAGAAGGAGTGGCTAAG
	**R**	TCTGACCACAGTGAGGAATGTCCAC
tnf-a	**F**	ATAGCTCCCAGAAAAGCAAGC
	**R**	CACCCCGAAGTTCAGTAGACA
ifn-g	**F**	ACTCAAGTGGCATAGATGTGGAAG
	**R**	GACGCTTATGTTGTTGCTGATGG
il-17	**F**	TCCAGAAGGCCCTCAGACTA
	**R**	AGCATCTTCTCGACCCTGAA
il-1b	**F**	GCCTTGGGCCTCAAAGGAAAGAATC
	**R**	GGAAGACACAGATTCCATGGTGAAG
Mip1a	**F**	CCCAGCCAGGTGTCATTTTCC
	**R**	GCATTCAGTTCCAGGTCAGTG
cxcl10	**F**	CATGGTCCTGAGACAAAAGT
	**R**	TGATGACACAAGTTCTTCCA
il10	**F**	GCCAGAGCCACATGCTCCTA
	**R**	GATAAGGCTTGGCAACCCAAGTAA
il5	**F**	TGAGGCTTCCTGTCCCTACTCATAA
	**R**	TTGGAATAGCATTTCCACAGTACCC
cox2	**F**	TCTGGAACATTGTGAACAACATC
	**R**	AAGCTCCTTATTTCCCTTCACAC
tlr2	**F**	CTCCTGAAGCTGTTGCGTTAC
	**R**	GCTCCCTTACAGGCTGAGTTC
tlr4	**F**	TCGCCTTCTTAGCAGAAACAC
	**R**	GCCTTAGCCTCTTCTCCTTC
foxp3	**F**	GAGAGGCAGAGGACACTCAATG
	**R**	GCTCAGGTTGTGGCGGATG
16s rRNA	**F**	TAAAGGCTCACCAAGGCGAC
	**R**	TTAGCCGGTGCTTTAGGCAG
Lipl32	**F**	AAGCATTACCGCTTGTGGTG
	**R**	GAACTCCCATTTCAGCGATT
IFN-g	**F**	GGCCATCCAGAGGAGCATAG
	**R**	TTTCTCCATGCTGCTGTTGAA
IL-4	**F**	CCACGGAGAAAGACCTCATCTG
	**R**	GGGTCACCTCATGTTGGAAATAAA
